# Changes in expression levels of Nod-like receptors in the spleen of ewes

**DOI:** 10.1590/1984-3143-AR2022-0093

**Published:** 2023-05-15

**Authors:** Jiaxuan Wu, Shengya Fang, Pengfei Feng, Chunjiang Cai, Leying Zhang, Ling Yang

**Affiliations:** 1 Department of Animal Science, School of Life Sciences and Food Engineering, Hebei University of Engineering, Handan, China

**Keywords:** Nod-like receptor, pregnancy, sheep, spleen

## Abstract

Nucleotide-binding oligomerization domain receptors (NOD-like receptors, NLRs) have critical effects on interfaces of the immune and reproductive systems, and the spleen plays a key role in both innate and adaptive immune functions. It is hypothesized that NLR family participates in maternal splenic immune regulation during early pregnancy in sheep. In this study, maternal spleens were collected on day 16 of the estrous cycle, and days 13, 16 and 25 of gestation (n = 6 for each group) in ewes. Expression of NLR family, including NOD1, NOD2, class II transactivator (CIITA), NLR family apoptosis inhibitory protein (NAIP), nucleotide-binding oligomerization domain, Leucine rich repeat and Pyrin domain containing 1 (NLRP1), NLRP3 and NLRP7, was analyzed using quantitative real-time PCR, Western blot and immunohistochemistry analysis. The results revealed that expression levels of NOD1, NOD2, CIITA and NLRP3 were downregulated at days 13 and 16 of pregnancy, but expression of NLRP3 was increased at day 25 of pregnancy. In addition, expression values of NAIP and NLRP7 mRNA and proteins were improved at days 16 and 25 of pregnancy, and NLRP1 was peaked at days 13 and 16 of pregnancy in the maternal spleen. Furthermore, NOD2 and NLRP7 proteins were limited to the capsule, trabeculae and splenic cords. In summary, early pregnancy changes expression of NLR family in the maternal spleen, which may be related with the maternal splenic immunomodulation during early pregnancy in sheep.

## Introduction

The maternal immunological adaptations are essential for a positive pregnancy, and deficient immunological tolerance leads to preeclampsia or miscarriage (Genebrier and Tarte, 2021). There are immune-mediated mechanisms of recurrent pregnancy loss, and immunotherapy is effective for the population selected for a poor prognosis, or immune phenomena to modulate these mechanisms and improve the live birth rate (Carp, 2019). Mammalian pregnancy induces unique changes in maternal immune systems, and pregnancy recognition factor (interferon-tau, IFNT), progesterone, pregnancy associate glycoproteins and chaperonin 10 are involved in altering immune function both locally and systemically during pregnancy in ruminants (Ott, 2020). IFNT modulates the maternal innate immune system and prevents conceptus rejection, and the pattern recognition receptors also work in parallel with IFNT to do it during early pregnancy in ruminants (Rocha et al., 2021). Our previous studies report that IFNT and progesterone have effects on expression of interferon-stimulated genes and progesterone receptors in the ovine bone marrow (Yang et al., 2017a; Zhang et al., 2017), thymus (Zhang et al., 2018; 2020b), spleen (Yang et al., 2018a; 2018b; Wang et al., 2019) and lymph nodes (Yang et al., 2017b; 2019; Zhang et al., 2020a) during early pregnancy.

The spleen interacts with the circulatory, reticuloendothelial and immune systems, and plays a key role in both innate and adaptive immune function (Crane et al., 2021). Cytotoxic T-lymphocyte antigen-4 and T-cell immunoglobulin mucin-3 are important negative immune regulatory molecules that are upregulated in splenic T cells, which are implicated in peripheral immune modulation and maternal tolerance during normal pregnancy (Wang et al., 2021). Interleukin-21 (IL-21) is a pro-inflammatory cytokine associated with altered immune responses, and splenic B cells from the mice with favorable pregnancy outcome express lower IL-21 receptor level than the mice with immune-induced bad pregnancy outcome (Fröhlich et al., 2020). During early pregnancy in sheep, expression levels of gonadotropin releasing hormone, prolactin and its receptor, T help cytokines, melatonin receptors and CD4 are modulated in the maternal spleen (Cao et al., 2021; Li et al., 2019b; Bai et al., 2020; Feng et al., 2022). In addition, nuclear factor kappa B signaling pathway, complement signaling pathway and Toll-like receptor signaling pathway are regulated by early pregnancy in the spleen of ewes (Hao et al., 2022; Yang et al., 2021; Zhang et al., 2021). Therefore, early pregnancy modulates splenic immune related signaling pathways.

Nucleotide-binding oligomerization domain receptors (NOD-like receptors, NLRs) are intracellular proteins that have their critical functions in inflammatory responses and the interfaces of the immune and reproductive systems (Van Gorp et al., 2014). NOD1 and NOD2 are increased in laboring fetal membranes and myometrium, which modulate proinflammatory and prolabor mediators in fetal membranes and myometrium via nuclear factor-kappa B (NF-κB) in humans (Lappas, 2013). Class II transactivator (CIITA) is a transcriptional coactivator, and the founding member of NLR protein family, plays a critical role in immune responses (Devaiah and Singer, 2013). The NLR family apoptosis inhibitory protein (NAIP) is important for mounting an immune response, and related to autoinflammatory disorders in humans (Sundaram and Kanneganti, 2021). NLRP (nucleotide-binding oligomerization domain, Leucine rich repeat and Pyrin domain containing) family, also referred to as NALP family, is expressed in human gametes and preimplantation embryos at different developmental stages (Zhang et al., 2008). However, it is unclear for the pattern of expression of NLR family, including NOD1, NOD2, CIITA, NAIP, NLRP1, NLRP3 and NLRP7, in the spleen of ewes during early pregnancy. In this study, the objective is to explore the expression of NLR family, which will be beneficial for understanding the maternal splenic immunomodulation during early pregnancy in ruminants.

## Methods

All of the animal-related procedures performed in this study were approved by the Hebei University of Engineering Animal Care and Use Committee (approval no. 2019-017).

### Animal tissue collection

The experiments were performed on ewes (Small-tail Han sheep) with approximately 18 months old and average weight of 41 kg, and housed at a farm in Handan, China. The females were fed twice a day with conventional breeding and nutrition, and kept indoors under natural lighting condition with free access to water and mineral licks. The ewes with normal estrous cycles were randomly divided into three pregnant groups (at pregnancy days 13, 16, and 25) and one nonpregnant group (day 16 of the estrous cycle) for collection of spleen tissue samples (n = 6 for each group). The ewes were observed daily for estrus in the presence of vasectomized rams. After detection of sexual receptivity (day 0 of pregnancy or nonpregnancy), the ewes were mated naturally for three pregnancy groups of pregnant ewes within half of month. For the nonpregnant group, ewes were not mated naturally. The animals were slaughtered, and spleens were sampled on days 13, 16, and 25 of pregnancy, and day 16 of the estrous cycle, and pregnancy was verified by observing an embryo in the uterus. The spleen samples were rinsed in phosphate-buffered saline (PBS), snap-frozen in liquid nitrogen, and stored at -80 °C until use for subsequent quantitative real-time PCR (qRT-PCR) and western blot analysis. In addition, some splenic samples were immediately fixed in fresh 4% (w/v) paraformaldehyde for following immunohistochemistry analysis.

### RNA extraction and qRT-PCR assay

Splenic tissues were homogenized in liquid nitrogen, and total RNA was obtained using TRIzol reagent (Invitrogen, California, USA) following the manufacturer’s instructions. Then, RNA (approximately 1 µg) was reverse transcribed into cDNA, and genomic DNA was eliminated using a FastQuant RT kit with DNase (Tiangen Biotech Co., Ltd., Beijing) according to the manufacturer’s instructions. Specific primers corresponding to the target genes and reference gene were designed and synthesized by Shanghai Sangon Biotech Co., Ltd. (Table 1). The cDNA was amplified using a SuperReal PreMix Plus kit (Tiangen Biotech) on a Bio-rad CFX96 real-time PCR system (Bio-Rad Laboratories, Inc., CA, USA). Amplification was performed under the following conditions: 95 °C for 10 minutes followed by 40 cycles of denaturation (95 ˚C for 10 s), annealing (59 to 62 °C for 20 s) and extension (72 °C for 25 s) followed by one cycle of final extension (72 ˚C for 7 minutes). The annealing temperatures were 60.5 ˚C for NOD1 and CIITA, or 62 ˚C for NOD2, or 59.5°C for NAIP, or 60 °C for NALP1, or 59°C for NLRP3, or 61°C for NLRP7. In addition, GAPDH was amplified in parallel with the target genes. The mean mRNA expression level for each target gene in each sample was normalized to the expression of reference gene (GAPDH). The 2^-ΔΔCt^ analysis method (Livak and Schmittgen, 2001) was used to calculate relative expression values using the mean cycle threshold values collected from nonpregnant ewes as calibrators.

**Table 1:** Primers used for RT-qPCR.

**Gene**	**Primer**	**Sequence**	**Size (bp)**	**Accession numbers**
*NOD1*	Forward	CCTTGGCTGTCAGAGATTGGCTTC	94	XM_042248630.1
	Reverse	GCTTCTGGCTGTATCTGCTCACTG		
*NOD2*	Forward	TGCCATCCTCGCTCAGACATCTC	117	XM_042231601.1
	Reverse	CAGCCACACTGCCCTCTTTGC		
*CIITA*	Forward	GCACCTCCTTCCAGTTCCTTGTTG	119	XM_042239890.1
	Reverse	CCTGTCCCAGTCCCTGAGATCG		
*NAIP*	Forward	TTGTCCAGCAGTGTCAGCATCTTC	82	XM_012096791.3
	Reverse	ATTTCCACCACGCTGTCATCATCC		
*NLRP1*	Forward	AAGGAGGTGACCGAGATGCTGAG	143	XM_012185551.4
	Reverse	TGCCGCTTGAGTGAGGATGTATTG		
*NLRP3*	Forward	CTCTGGTTGGTCAGTTGCTGTCTC	81	XM_042250402.1
	Reverse	GGTCAGGGAATGGTTGGTGCTTAG		
*NLRP7*	Forward	GCCTGCTACTCGTTCATCCATCTC	90	XM_004015893.5
	Reverse	CCCTTCCTCCTCCTGCTCTTCC		
*GAPDH*	Forward	GGGTCATCATCTCTGCACCT	176	NM_001190390.1
	Reverse	GGTCATAAGTCCCTCCACGA		

### Western blot

A RIPA lysis luffer (Biosharp, BL504A) was used to homogenize the splenic samples, and protein concentration was determined using a BCA protein assay kit (Tiangen Biotech). Total proteins (10 μg) were fractionated by electrophoresis on 12% SDS polyacrylamide gels, and then transferred from the gel onto a polyvinylidene fluoride membrane (Millipore, Bedford, MA, USA) by electroblotting. The membranes were blocked with 5% nonfat dry milk at 4 °C overnight, and then were incubated with a mouse anti-NOD1 monoclonal antibody (Santa Cruz Biotechnology, Santa Cruz, CA, USA, sc-398696), a mouse anti-NOD2 monoclonal antibody (Santa Cruz Biotechnology, sc-56168), a mouse anti-CIITA monoclonal antibody (Santa Cruz Biotechnology, sc-13556), a rabbit anti-NAIP polyclonal antibody (Abcam, Cambridge, UK, ab25968), a mouse anti-NLRP1 monoclonal antibody (Santa Cruz Biotechnology, sc-390133), a mouse anti-NLRP3 monoclonal antibody (Santa Cruz Biotechnology, sc-134306), and a mouse anti-NLRP7 monoclonal antibody (Santa Cruz Biotechnology, sc-377190) at a dilution of 1:1000, respectively. After washed three times, the membranes were incubated with horseradish peroxidase-conjugated secondary antibody (goat anti-mouse IgG, Biosharp, BL001A; or goat anti-rabbit IgG, Biosharp, BL003A) at a dilution of 1:10000. Immunoreactive bands were detected using an ECL western blotting detection reagent (Tiangen Biotech). Equal amounts of sample protein were loaded onto the gels, and equal exposure times were used for the processing and development of the Western blots using an anti-GAPDH antibody (Santa Cruz Biotechnology, Inc., sc-20357). The immunospecific bands were quantified using with GAPDH as an internal control protein using an anti-GAPDH antibody (Santa Cruz Biotechnology, sc-20357, 1:1000). Data were analyzed by Quantity One V452 (Bio-Rad Laboratories, Hercules, CA, USA), and normalized to GAPDH for loading correction.

### Immunohistochemistry analysis

For immunohistochemistry, fixed splenic tissues were embedded in paraffin, and then cut to 5 μm-thick sections. After routine rehydration and antigen retrieval, some sections were stained for hematoxylin and eosin (HE). Endogenous peroxidase was quenched using 3% H_2_O_2_, and nonspecific binding was blocked in 5% normal goat serum. Immunohistochemical localization of NOD2 and NLRP7 in the splenic tissue was performed using the mouse anti-NOD2 monoclonal antibody (Santa Cruz Biotechnology, sc-56168) or the mouse anti-NLRP7 monoclonal antibody (Santa Cruz Biotechnology, sc-377190) at 1:200 dilution. After being rinsed with PBS, tissue sections were incubated with anti-mouse secondary antibody (Biosharp, BL001A). Negative control slides were run simultaneously with no primary antibody. The sections were rinsed with PBS, and positive labeling was visualized using a DAB kit (Tiangen Biotech), and then nuclear was stained with hematoxylin. Images were acquired on a light microscope (Nikon Eclipse E800, Japan) with a digital camera (AxioCam ERc 5s). The intensity of staining and density of the stained cells were analyzed through the images independently by 4 observers in a blinded fashion. Staining intensities were analyzed by assigning an immunoreactive intensity of a scale of 0 to 3, as described previously (Li et al., 2019b).

### Statistical analyses

Data for relative expression levels of NOD1, NOD2, CIITA, NAIP, NLRP1, NLRP3 and NLRP7 mRNA and proteins were presented as means ± SE, and analyzed with MIXED procedure in SAS (Version 9.1; SAS Institute, Cary, NC). When a statistical difference was observed, data were further analyzed using Bonferroni’s post hoc correction. Duncan method was used to compare the relative expression levels of the different groups, and controlling the experimentwise type ± error equal to 0.05. Differences were statistically significant when *P* < 0.05.

## Results

### Expression of NOD1, NOD2, CIITA, NAIP, NLRP1, NLRP3 and NLRP7 mRNA in the spleen

**Figure 1:**
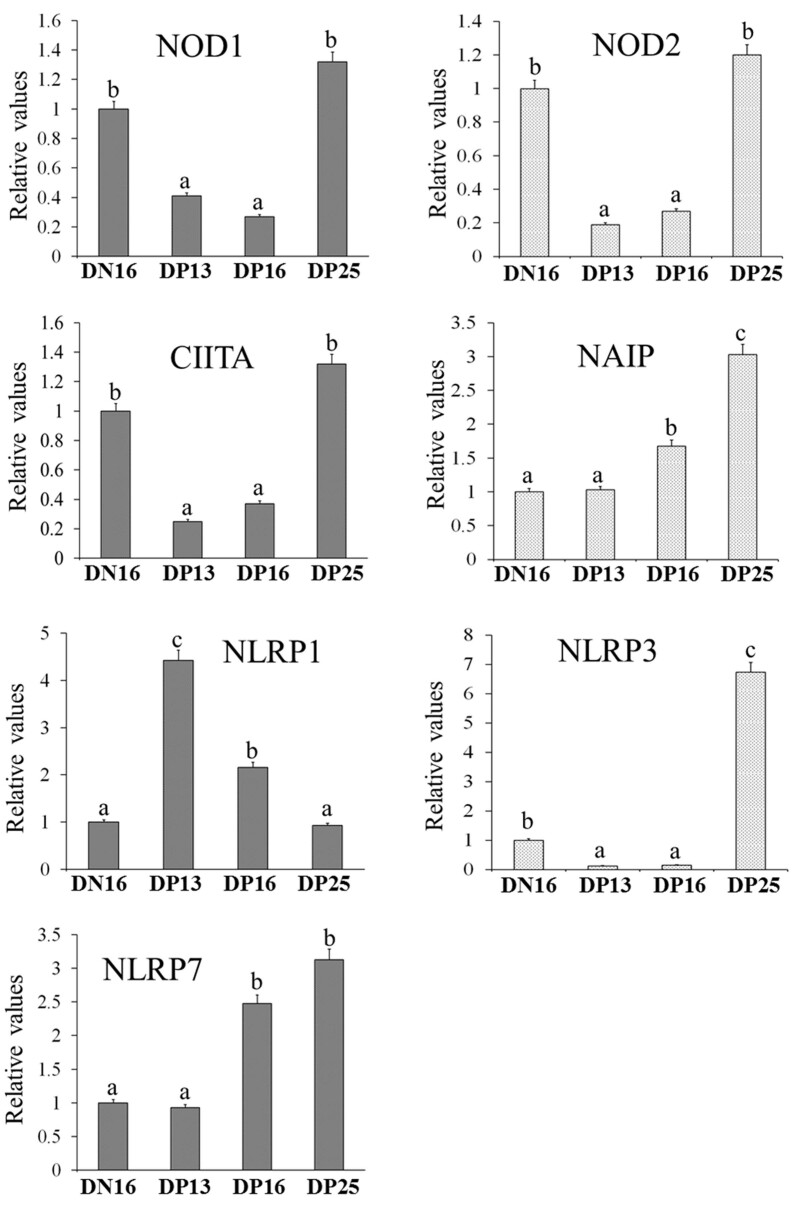
Relative expression values of *NOD1, NOD2, CIITA, NAIP, NLRP1, NLRP3 and NLRP7* mRNA in ovine spleens measured by quantitative real-time PCR. Note: DN16 = day 16 of the estrous cycle; DP13 = day 13 of pregnancy; DP16 = day 16 of pregnancy; DP25 = day 25 of pregnancy. Significant differences (*P* < 0.05) are indicated by different letters within same color column.

On days 13 and 16 of gestation, there was a decrease in mRNA expression levels of *NOD1, NOD2*, *CIITA* and *NLRP3* compared to that at day 16 of the estrous cycle and day 25 of pregnancy (*P* < 0.05; Figure 1), and expression level of *NLRP3* mRNA was upregulated on day 25 of gestation. In addition, expression levels of *NAIP* and *NLRP7* were increased at days 16 and 25 of gestation compared to day 16 of the estrous cycle and day 13 of pregnancy (*P* < 0.05; Figure 1). Nevertheless, expression level of *NLRP1* was peaked at days 13 and 16 of pregnancy compared to day 16 of the estrous cycle and day 25 of pregnancy (*P* < 0.05; Figure 1).

### Expression of NOD1, NOD2, CIITA, NAIP, NLRP1, NLRP3 and NLRP7 proteins in the spleens

**Figure 2:**
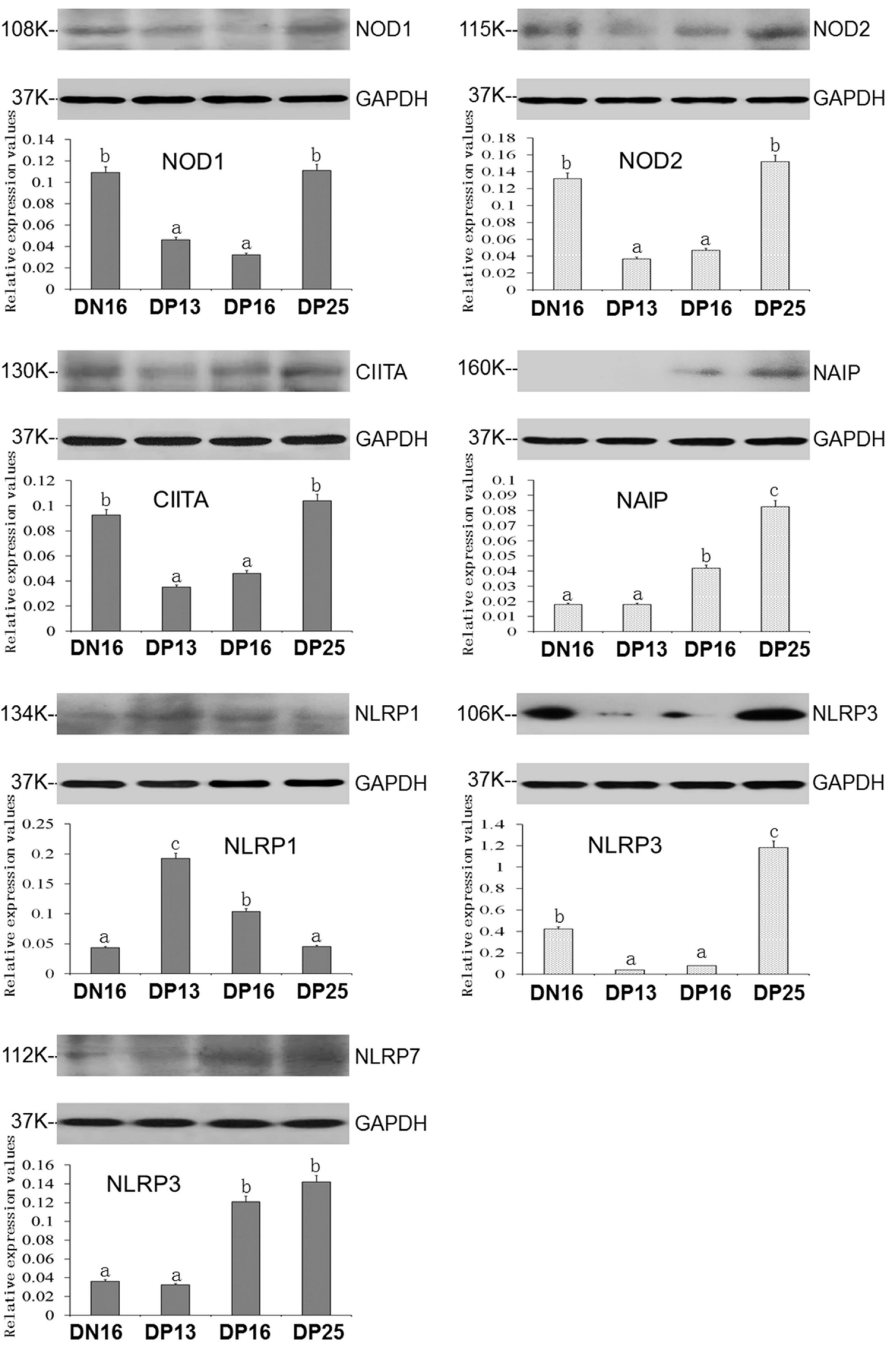
Expression of NOD1, NOD2, CIITA, NAIP, NLRP1, NLRP3 and NLRP7 proteins in ovine spleens analyzed by western blot. Note: DN16 = day 16 of the estrous cycle; DP13 = day 13 of pregnancy; DP16 = day 16 of pregnancy; DP25 = day 25 of pregnancy. Significant differences (*P* < 0.05) are indicated by different superscript letters within the same color column.

It was showed in Figure 2 that NOD1, NOD2, CIITA and NLRP3 protein levels were lower on days 13 and 16 of gestation than that day 16 of the estrous cycle and day 25 of pregnancy (*P*< 0.05), and protein expression level of NLRP3 was the highest at day 25 of pregnancy among these four groups in the spleen. Early pregnancy induced expression of NAIP and NLRP7 proteins at days 16 and 25 of pregnancy compared to day 16 of the estrous cycle and day 13 of pregnancy (*P* < 0.05), and NAIP protein was almost undetected at day 16 of the estrous cycle and day 13 of pregnancy. However, expression level of NLRP1 protein was upregulated at days 13 and 16 of pregnancy compared to that at day 16 of the estrous cycle and day 25 of pregnancy (*P* < 0.05; Figure 2).

### Immunohistochemistry for NOD2 and NLRP7 proteins in the spleens

**Figure 3:**
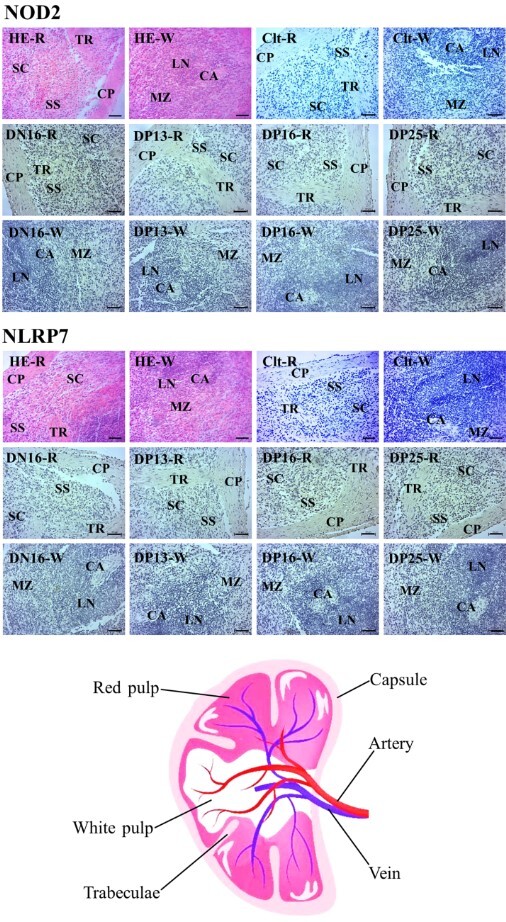
Representative immunohistochemical localization of NOD2 and NLRP7 proteins in ovine spleens and a schematic drawing of the spleen. The spleen is divided into red pulp (R) and white pulp (W), and surrounded by a thickened capsule. Capsule (CP) with several trabeculae (TR) projects into the substance of the spleen. Note: HE = stained by hematoxylin and eosin; Clt = negative control; SS = splenic sinuses; SC = splenic cords; MZ = marginal zone; LN = lymphoid nodule; CA = central arteriole; DN16 = day 16 of the estrous cycle; DP13 = day 13 of pregnancy; DP16 = day 16 of pregnancy; DP25 = day 25 of pregnancy. Bar = 50 µm.

NOD2 and NLRP7 proteins were located in the capsule, trabeculae and splenic cords. For the negative control, the spleens from day 16 of the estrous cycle and spleens from days 13, 16 and 25 of pregnancy, the staining intensities for NOD2 protein were 0, 2, 1, 1 and 2, respectively (Figure 3), while the staining intensities for NLRP7 protein were 0, 1, 1, 2 and 2, respectively (Figure 3). The staining intensity was as follows: 0 = negative; 1 = weak; 2 = strong.

## Discussion

NOD1 is a pattern recognition receptor, and expressed by immune and non-immune cells, which contributes to both immune memory and tolerance (Fernández-García et al., 2021). Recurrent pregnancy loss is related to NOD1 aberrant expression in decidual stromal cells (DSCs), and NOD1 expression in DSCs is essential for maintaining pregnancy via regulation of immune responses (Zhang et al., 2019). Expression level of NOD1 is higher in trophoblasts than that in decidua and placenta, which plays a role in direct maternal-fetal communication in normal pregnancy (Rakner et al., 2021). *NOD1* mRNA is upregulated in an *in vitro* model of decidualization of endometrial stromal cell, which is associated with expression of innate immune molecules in endometrium, and has a role in innate immune protection in the uterus (King et al., 2009). Splenic NOD1 activation reduces macrophage recruitment, and plays a key role in iron homeostasis that is necessary for central biological functions in mice (Fernández-García et al., 2022). In this study, expression of NOD1 was downregulated on days 13 and 16 of pregnancy, but upregulated on day 25 of pregnancy in the maternal spleen. Therefore, the expression of NOD1 was modulated in the maternal spleen during early pregnancy, which may be associated with regulation of splenic immune response in sheep.

NOD2 plays key roles in T helper 17 (Th17) cell differentiation and T cell homeostasis, which contribute to immune tolerance (Napier et al., 2018). NOD2 expression in DSCs plays an important role in protecting the embryo and preventing infection in the maternal-fetal interface through modulation of maternal innate immune responses during the first trimester of pregnancy (Zhang et al., 2014b). There is significantly higher mRNA and protein levels of NOD2 in DSCs from the normal pregnancy group than the unexplained recurrent spontaneous abortion group, suggesting that NOD2 is necessary for sustaining normal pregnancy in humans (Zhang et al., 2014a). The syncytiotrophoblast and cytotrophoblast cells express NOD2 in first trimester, which is important owing to lack the transmembrane TLRs in these cells for controlling infections at the maternal-fetal interface (Abrahams, 2008). NOD2 activation via systemic administration of muramyl dipeptide contributes to an increase in splenic regulatory T cells, which results in immunological tolerance (Prescott et al., 2020). Our results revealed that NOD2 mRNA and protein downregulated at days 13 and 16 of pregnancy, but upregulated in the maternal spleen at day 25 of pregnancy. NOD2 protein was located in the capsule, trabeculae and splenic cords. Therefore, modulation of NOD2 may be helpful for modulation of maternal innate immune responses of the maternal spleen during early pregnancy.

CIITA is the founding member of NLR protein family, and the master regulator of MHC class II gene expression that contributes to intrinsic immunity in mammals (León Machado and Steimle, 2021). Knockdown of CIITA expression has no effect on cleavage of *in vitro*-derived embryos, but disrupts development of embryos into the blastocyst stage, which suggesting that CIITA plays important roles in bovine preimplantation development (Nagatomo et al., 2015). CIITA protein is expressed in a cell type- and pregnancy status-specific manner in the endometrium during the implantation period in pigs, which is regulated by interferon-γ and related to the establishment of pregnancy in pigs (Yoo et al., 2020). There is a lower expression level of IFN-γ in the maternal spleen at days 13 and 16 of pregnancy comparing to day 16 of the estrous cycle and day 25 of pregnancy (Li et al., 2019b). In this study, expression level of CIITA in the maternal spleen is also lower at days 13 and 16 of pregnancy than day 16 of the estrous cycle and day 25 of pregnancy. Therefore, the downregulation of CIITA at days 13 and 16 of pregnancy may be associated with the decrease of IFN-γ in the maternal spleen, which may be related to the splenic immune modulation at implantation period in sheep.

As a member of NLR superfamily, NAIP also is a member of the inhibitor of apoptosis protein family, and has anti-apoptotic and innate immunology functions in mammalians (Abadía-Molina et al., 2017). NAIP protein is strongly expressed in first trimester placentas comparing to that in the term placentas, which plays critical roles in placental cell survival (Ka and Hunt, 2003). Long non-coding RNA AK002210 modulates the expression of NAIP, which regulates the phenotype of the trophoblast cell, and plays a critical role in the progression of preeclampsia (Zhao et al, 2020). *NAIP* mRNA is present in the developing mouse embryos, which is involved in the embryogenesis in mice (Ingram-Crooks et al., 2002). *NAIP* mRNA transcript is expressed in macrophage-rich tissues, including the spleen, and NAIP protein expression is upregulated after phagocytic events (Diez et al., 2000). It is found that expression of *NAIP* mRNA and protein was enhanced in the maternal spleen at days 16 and 25 of pregnancy in this study. Therefore, the upregulation of NAIP may be related to the regulation of maternal splenic functions, and helpful for pregnancy establishment.

NLRP1 is expressed by several cell types, specifically, and acts as a negative regulator in T helper 17 (Th17) differentiation (Costa et al., 2021). Th17 cell is an active player in the establishment of tolerance and defence, and Treg cells dominate Th17 cells to guarantee fetal survival during pregnancy (Figueiredo and Schumacher, 2016). Expression level of NLRP1 is negatively correlated with expression levels of glutathione and glutathione peroxidase 4, which are related to placental dysfunction and adverse pregnancy (Meihe et al., 2021). Preeclampsia is associated with abnormal activation of cells from the innate immune system, and the peripheral blood monocytes from preeclamptic pregnant women have higher endogenous activation of NLRP1/NLRP3 inflammasomes than that from normotensive pregnant women (Matias et al., 2019). There is an upregulation of NLRP1 in a placental trophoblast oxidative stress model, and resveratrol ensures the normal biological functions of trophoblasts with a downregulation of NLRP1 (Li et al., 2020). Our data showed that there was a downregulation of NLRP1 mRNA and protein in the maternal spleen at day 25 of pregnancy. Therefore, downregulation of NLRP1 may be related to the establishment of immune tolerance in the maternal spleen, and necessary for normal pregnancy.

NLRP3 inflammasome is a multiprotein complex that orchestrates innate immune responses to infection and cell stress, and NLRP3 inflammasome is activated in response to diverse stimuli (Elliott and Sutterwala, 2015). Uric acid, or monosodium urate, activates NLRP3 to lead to inflammasome activation and IL-1β processing, which results in induction of inflammation at the maternal-fetal interface, leading to placental dysfunction and adverse pregnancy outcome (Mulla et al., 2011). NALP3 inflammasome senses danger signals to result in the production of IL-1β, but IL-1β not NALP3 inflammasome is an important determinant of endothelial cell responses to necrotic/dangerous trophoblastic debris (Wei et al., 2015). Edoplasmic reticulum (ER) has a key role in decidualization and placentation, and ER stress activates/triggers sensing proteins to induce activating the NLRP3 inflammasome that is in favor of in decidualization and placentation (Soczewski et al., 2020). Our results revealed that expression level of NLRP3 was downregulated at days 13 and 16 of pregnancy, but upregulated at day 25 of pregnancy. Therefore, downregulation of NLRP3 may be related to the immune regulation in the maternal spleen at days 13 and 16 of pregnancy, and upregulation at day 25 of pregnancy may be not related to placentation.

NLRP7 is related to innate immune signaling, and has positive and negative effects on inflammasome responses, and NLRP7 mutations contribute to reproductive diseases (Carriere et al., 2021). NLRP7 increases trophoblast proliferation, and its expression ensures a tolerance of the trophoblast by the maternal immune system via regulation of key immune tolerance-associated factors during normal pregnancy (Abi Nahed et al., 2022). NLRP7 contributes to *in vitro* decidualization of endometrial stromal cells that is a hallmark of tissue remodeling to support embryo implantation and proper placental development (Huang et al., 2017). NLRP7 mutation leads to recurrent hydatidiform moles in humans, and NLRP7 protein is mainly located in ovine ovarian follicles and in *in vitro* preimplantation embryos, which plays an important role in ovine reproduction (Li et al., 2019a). In this study, the NLRP7 mRNA and protein were increased at days 16 and 25 of pregnancy in the maternal spleen, and NLRP7 protein was located in the capsule, trabeculae and splenic cords. Therefore, the upregulation of NLRP7 at days 16 and 25 of pregnancy may contribute to the immune tolerance, and be essential for embryo implantation in ewes.

## Conclusion

Early pregnancy suppressed expression of NOD1, NOD2, CIITA and NLRP3 at days 13 and 16 of pregnancy, but enhanced expression of NLRP3 at day 25 of pregnancy. Furthermore, there was an upregulation of NAIP and NLRP7 mRNA at days 16 and 25 of pregnancy, and NLRP1 was increased at days 13 and 16 of pregnancy in the maternal spleen. In addition, NOD2 and NLRP7 proteins were located in the capsule, trabeculae and splenic cords. There may have some relationship between expression of NLR family in the fetal tissues, the placenta, and in the maternal spleen. Therefore, early pregnancy modulated the expression of NLR family in the maternal spleen, which may be associated with regulation of maternal splenic immune response in sheep.
